# Xanthine oxidoreductase activity in platelet-poor and rich plasma as a oxidative stress indicator in patients required renal replacement therapy

**DOI:** 10.1186/s12882-021-02649-8

**Published:** 2022-01-18

**Authors:** Elżbieta Cecerska-Heryć, Rafał Heryć, Grażyna Dutkiewicz, Anna Michalczyk, Bartłomiej Grygorcewicz, Natalia Serwin, Sylwia Napiontek-Balińska, Barbara Dołęgowska

**Affiliations:** 1 Department of Laboratory Medicine, Pomeranian Medical University in Szczecin, Powstanców Wielkopolskich 72, 70-111 Szczecin, Poland; 2 Department of Nephrology, Transplantology and Internal Medicine, Pomeranian Medical University in Szczecin, Powstancow Wielkopolskich 72, 70-111 Szczecin, Poland; 3 Department of Psychiatry, Pomeranian Medical University in Szczecin, Broniewskiego 26, 71-460 Szczecin, Poland

**Keywords:** Xanthine oxidoreductase, Platelets, Renal replacement therapy, Chronic kidney disease, Antioxidant enzymes

## Abstract

**Background:**

Xanthine oxidoreductase (XOR) is a hydroxylase enzyme involved in the metabolism of purines. XOR activity can vary: the homodimer protein can be converted into two different isoforms XD (antioxidant) and XO (prooxidant). Oxidative stress and inflammation that accompanying chronic kidney disease (CKD), dialysis, and kidney transplantation, resulted in platelet activation. Present study aimed to determine the influence of applied renal replacement therapy on xanthine oxidoreductase and its isoforms activity.

**Materials and Methods:**

The study group consisted of 117 patients, divided into 4 groups: hemodialysis - 30 patients, peritoneal dialysis - 30 patients, kidney transplant patients - 27 and conservative treatment - 30 patients. The control group consisted of 30 healthy volunteers.

**Results:**

Significant differences were found in XOR activity in platelet-poor plasma (PPP) within the groups studied (*p* = 0.001). There was a relationship between the type of renal replacement therapy of all oxidoreductase isoforms in PPP (*p* < 0.001 all isoforms) and XD (*p* = 0.008), XO (*p* < 0.001) in platelet rich-plasma (PRP). A relationship was observed between the activity of all oxidoreductase isoforms in PPP and PRP, and the type of renal replacement therapy and the duration of dialysis and the age of patients. The cause of chronic kidney disease was also reflected differences in XD and XO activity in PPP.

**Conclusions:**

The type of renal replacement therapy used in CKD patients, age of patients, duration of dialysis, CKD causes, and stage of progression significantly affect the activity of XOR and its isoforms.

## Background

Xanthine oxidoreductase (XOR) is a hydroxylase enzyme involved in the metabolism of purines. It catalyzes the oxidation of hypoxanthine to xanthine and xanthine to uric acid (UA). The XOR activity can vary: the homodimer protein can be converted into two different isoforms. Xanthine dehydrogenase (XD) expressed predominantly in healthy tissue, and xanthine oxidase (XO) generated by post-translational XD modification, oxidation cysteine residues as well as limited proteolysis, playing a dominant role in cells and tissues during injuries [1–3]. The activities of the mentioned isoforms oppose each other [4]. XOR acts in the presence of NAD^+^ as a dehydrogenase, and with molecular oxygen as an oxidase. The ability of XOR to rapidly convert from antioxidant to oxidant, in various types of tissue damage, is an essential element for a rapid innate immune response, beneficial in, for example, in bacterial or fungal infection [5].

A reaction intermediates of xanthine dehydrogenase (XDO) and oxygen can react with both NAD^+^ and O_2_, but present a higher affinity for NAD^+^ [6]. This intermediate isoform has not been isolated, but the determination of its activity facilitates the tracking of the transformation of XD to XO [7].

The serum XOR activity in various diseases has been widely investigated. The reason of the interest in this enzyme is the dualism of its action: the ability to produce antioxidants, and on the other hand, reactive oxygen species creation. An increase in XOR activity occurs in pathological conditions such as viral hepatitis, infectious mononucleosis, autoimmune diseases, pneumonia, schizophrenia, and type II diabetes. The increase in XOR activity is also observed in the serum of patients after renal or liver transplantation [8]. It has been shown that the gene coding for XOR may be responsible for renal maturation, adipogenesis in the kidneys, and may prevent the transformation of epithelial cells into mesenchymal tissue [9]. However, due to our best knowledge, there are no published reports investigating XOR activity in platelet-poor plasma (PPP) and platelet-rich plasma (PRP) in patients undergoing renal replacement therapy. The importance of the XOR activity in this group of patients is related to increased platelet activation, which is caused by the oxidative stress and inflammation that accompany chronic kidney disease (CKD), dialysis, and kidney transplantation. Besides, during dialysis and organ transplantation, tissues and blood vessels are damaged, and platelets are the first cells to reach the site of tissue damage, actively participating in the initial stages of the inflammatory process and healing [10].

PRP and PPP are fractions of blood plasma with different platelet concentrations. The platelet content of PRP and PPP is platelets/ml and platelets/ml, respectively. PPP and PRP are obtained by repeatedly centrifuging and washing the whole blood of humans at different centrifugal speeds [11, 12].

PPP, as a centrifugation byproduct of anticoagulated blood, has a lower platelet concentration than normal blood. The main components of PPP are fibrinogen, fibronectin, and thrombin. The biological effects of PPP are participating in hemostasis and coagulation, acting as a cell attachment vector, and promoting mitosis of fibroblasts and epithelial cells [13]. Although PPP is not as concentrated in platelets as PRP, it has been demonstrated that PPP can also sustain cell growth and survival. PPP promotes wound healing-associated cell functions and accelerates cell migration and proliferation of fibroblasts [14, 15].

Platelet-rich plasma has more concentrated platelets than normal plasma (approximately 150–400 × 10^3^ cell/dL). It is one of the most common definition of PRP in the literature [16].

Due to date, limited studies characterized XOR and its isoforms activity in platelet-rich or poor plasma in patients suffering from chronic kidney disease. Tan *et al.* showed cells damaged by reactive oxygen species (ROS) (in the case in chronic renal replacement therapy) “leak” XOR isoforms, leading to the increasement of the enzyme level in plasma [17]. This mechanism explains the lower activity of XOR and its isoforms in platelets compared to PPP. Therefore, the oxidoreductases activity in PPP and PRP is of great interest, as it could help to uncover the cellular processes that occur during dialysis. Such investigations could also indirectly highlight the severity of oxidative stress in this group of patients.

Berry et al. describe also that xanthine oxidoreductase is distributed in the liver, small intestine, mammary gland, and endothelial cells. Subcellular localization methods have demonstrated the presence of xanthine oxidoreductase both in the cytoplasm and on cell membranes. They also indicate that plasma xanthine oxidoreductase may be due to xanthine oxidoreductas shading from cell membranes or leaking from the cytoplasm. This is one of the reasons we test the activity of XOR in PPP and PRP to distinguish the enzyme activity in platelets and other blood cells contained in the plasma [18].

Based on the activity of antioxidant enzymes, we can also determine which type of renal replacement therapy is less likely to expose the patient to oxidative stress. Due to the dual nature of XOR, the understanding of the relationship between the type of renal replacement therapies and the activity of XOR isoforms can be very interesting and helpful in the selection of the type of renal replacement therapy.

## Materials and methods

### Ethical approval and consent

The Bioethical Commission at the Pomeranian Medical University in Szczecin approved the research carried out **(no KB − 0012/36/11**). All participants, including the healthy volunteers in the control group, were informed about the purpose and scope of the study and gave their consent to donate samples and publication of the resulting data.

### Study group

There were 147 participants: a control group of 30 healthy volunteers (NK), and 117 patients with chronic kidney disease (CKD) attending the Nephrology, Transplantology and Internal Diseases Clinic of the Pomeranian Medical University in Szczecin. The patients were divided into 4 groups based on the treatment they received: 30 patients before and after hemodialysis (HD A and HD B): 30 patients received peritoneal dialysis (PD); 27 patients before and after kidney transplantations (5–7 days after surgery) (TE, TE A); 30 patients received conservative treatment (CT) (CKD stage 2–5). The gender, age, duration of dialysis, cause and stage of chronic kidney disease, and creatinine concentration in the test and control groups are given in Tables 1 and 2.

**Table 1:** General characteristics of hemodialysis patients (HD), peritoneal dialysis (PD) treated conservatively (CKD), kidney transplantation (TE) and control group (C) participating in the study (mean ± SD)

Parameters	HD	PD	CKD	TE	NK	p*	p**
Gender [M– male; F – female]	M– 18 F– 12	M– 16 F– 14	M– 17 F– 13	M– 14 F– 13	M– 18 F– 12	NS	NS
Age [years]	63 ± 16	55 ± 15	66 ± 15	57 ± 11	50 ± 8	<0,001	0,029
Dialysis duration [months]	25 ± 16	26 ± 22	–	54 ± 34	–	–	0,003
Causes of CKD 1 – DM	5 (17%)	5 (17%)	4 (13%)	1 (4%)	–	–	NS
2 – HA	15 (50%)	3 (10%)	6 (20%)	0 (0%)	–	–	NS
3 – GIK	2 (7%)	9 (30%)	6 (20%)	3 (11%)	–	–	NS
4 – ADPKD	0 (0%)	0 (0%)	4 (13%)	2 (7%)	–	–	NS
5 – others	5 (17%)	10 (33%)	4 (13%)	6 (22%)	–	–	NS
6 – unknown	3 (10%)	3 (10%)	6 (20%)	15 (56%)	–	–	NS

*P* * - statistical significance for differences between HD, PD and CKD groups, TE and C exact Fisher test for qualitative variables; for quantitative variables - one-way ANOVA and; *P* ** - statistical significance for differences between HD, PD and CKD groups and TE exact Fisher test for qualitative variables for quantitative variables - one-way ANOVA or; *DM* - diabetic nephropathy; *HA* - hypertension; *GIK* - glomerular inflammation kidney; *ADPKD* - polycystic kidney disease inherited autosomal dominant; *NS* - no statistically significant differences.

**Table 2:** General characteristics of hemodialysis patients (B - before HD, A - after), peritoneal dialysis (PD) treated conservatively (CKD) before and after kidney transplantation (TE B and TE A) and control group (NK) taking part in the study (mean ± SD)

Parameters	HD B	HD A	PD	CKD	TE B	TE A	C	P*	P**
Kt/V	1,3 ± 0,2	–	2,8 ± 1,06	–	–	–	–	–	<0,001
Concentration of creatinine [mg/dl]	7,9 ± 2,4	3,5 ± 1,3	4,4 ± 2,2	2,5 ± 1,1	7,4 ± 3,3	3,4 ± 2,9	0,8 ± 0,1	<0,001	<0,001
Concentrations of uric acid [mg/dl]	4,5 ± 0,9	3,1 ± 0,67	5,7 ± 1,4	6,3 ± 1,3	7,2 ± 2,5	7,2 ± 1,8	6,9 ± 1,1	<0,001	<0,001
Concentration of albumin [g/dl]	3,3 ± 0,4	3,1 ± 0,3	3,9 ± 0,8	3,7 ± 0,8	3,8 ± 0,6	3,2 ± 0,5	3,8 ± 0,4	NS	NS
Concentration of phosphorum [g/dl]	1,8 ± 0,58	1,52 ± 0,44	1,55 ± 0,3	1,31 ± 0,3	1,81 ± 0,51	1,23 ± 0,38	–	–	NS
BMI	–	24,9 ± 3,9	26,2 ± 2,1	30 ± 2,9	–	26,3 ± 3,8	25,1 ± 3,6	NS	NS
Diabetes	–	N – 24 Y – 6	N – 25 Y – 5	N – 20 Y – 10	N – 26 Y – 1	–	N – 30 Y – 0	0,01	NS
Dyslipidemia	–	N – 26 Y – 4	N – 25 Y – 5	N – 14 Y – 16	N – 8 Y – 19	–	N– 30 Y– 0	0,01	NS
Stage of CKD:									
1	0 (0%)		0 (0%)	0 (0%)	0 (0%)		29 (97%)	–	–
2	0 (0%)	–	0 (0%)	3 (10%)	0 (0%)	–	1 (3%)	NS	NS
3	0 (0%)	–	0 (0%)	10 (33%)	0 (0%)	–	0 (0%)	NS	–
4	0 (0)%)	–	0 (0%)	12 (40%)	0 (0%)	–	0 (0%)	–	–
5	30 (100%)	–	31 (100%)	5 (17%)	27 (100%)	–	0 (0%)	–	NS

*P* * - statistical significance for differences between HD A, HD B, PD and CKD groups, TE and C for quantitative variables - Kruskal Wallis ANOVA, one-way ANOVA or Student’s t test

*P* ** - statistical significance for differences between HD A, HD B, PD and CKD and TE groups for Kruskal Wallis’s ANOVA quantitative variables or ANOVA one-way analysis

Kt / V - dialysis index (volume fraction V purified by clearance K at time t)

*NS* no statistically significant relationships were found

### Samples

Blood samples (K_2_EDTA (8 ml), 3.8% trisodium citrate (9: 1; v / v) and serum (8 ml)) were drawn from all study participants. Hemodialyzed patient blood was drawn from their arteriovenous fistula; peripheral venipuncture was used for all other participants. Samples were taken from hemodialysis patients before (HD A) and about 10 min after the pump was stopped (HD B). Transplant patient blood was collected before transplantation (TE) and 5–7 days after surgery (TE A). Patients recruited for the TE group did not belong to the group of hemodialysis or peritoneal dialysis patients in this study. They were patients qualified for transplantation from all over Poland. The vast majority of patients with kidney transplantation had prior hemodialysis. K_2_EDTA and clotted blood samples were centrifuged at 2600 rpm for 10 min at 20 °C to obtain plasma and serum, respectively. In order to obtain platelet-rich plasma (PRP) and platelet poor plasma (PPP), blood collected with citrate was centrifuged under the conditions at 1100 rpm for 10 min at 20 °C. The resulting PRP was transferred to a new tube and centrifuged at 6000 rpm for 10 min at 20 °C: platelet-poor plasma (PPP) was transferred to a separate tube; the platelet pellet was rinsed twice and suspended in Tyroda buffer (pH 7.4). Plasma, serum, PPP, and PRP were frozen at −80 °C until the assays were performed.

In hemodialysis patients, blood was collected before heparin administration and after dialysis lasting 4–5 h on average (heparin half-life - 4 h) to eliminate any possible influence of heparin on XOR activity.

### Xanthine oxidoreductase activity in platelet-poor plasma and platelets

Determinations were carried out with a Perkin Elmer UV/VIS Lambda 40P spectrophotometer. Extinction changes were recorded at 340 nm (XD) and 302 nm (XDO, XO) for 5 min at 30 °C. The enzymatic activity was measured as the formation of uric acid and NADH (increases in A_340_ and A_302_) and expressed in mU × mL^−1^ (milliunits per milliliter). The enzymatic activity was calculated, taking into account the initial rates of reaction. The uric acid formation was measured at 302 nm (isoforms XDO and XO) because its absorbance is still high there, whereas changes in NAD^+^ concentration do not contribute. The extinction coefficient for NADH^+^ H^+^ ε340 = 6.22 × 10^3^ [L∙mol^−1^ cm^−1^] was used to calculate the activity of isoforms of xanthine oxidoreductase NADH^+^ H^+^: ε302 = 2.30 × 10^3^ [L∙mol^−1^ cm^−1^] [7, 19–22].

### Statistical analysis

To assess distributions, the K-S test (Kolmogorov-Smirnov) was used, which in the case of some variables (the activity of XD and XDO isoforms in PRP) showed a non-normal distribution of parameters. Exact Fisher and Chi-square tests were used to analyze quantitative data. Using the Student’s t-test and ANOVA analysis for univariate systems, the differences between associated (paired) and unrelated (unpaired) variables were evaluated in the case of variables with a normal distribution. In the case of variables with non-normal distributions, Kruskal-Wallis ANOVA analysis was performed to evaluate differences between the parameters, as well as the Mann-Whitney U nonparametric test for unpaired data or Wilcoxon for paired data. A linear multiple regression model was used to determine the multifactor evaluation of relationships between the parameters studied. Statistical analysis of results was performed using Statistica 12 (StatSoft).

## Results

### The activity of xanthine oxidoreductase in poor and platelet-rich plasma

**Fig. 1:**
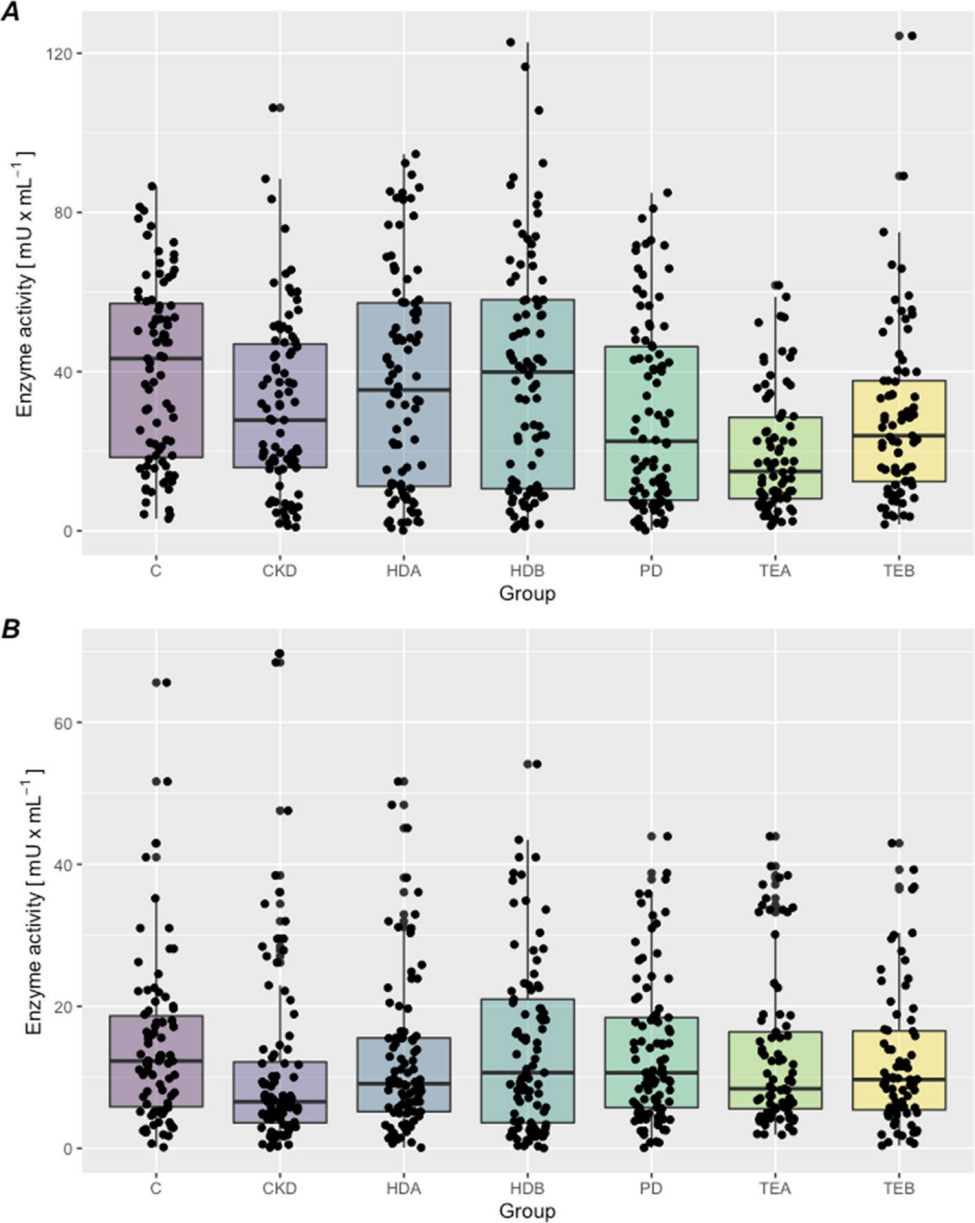
**A, B**. The xanthine oxidoreductase isoforms activity: **A** in platelet-rich plasma. **B** in platelet-poor plasma. C- control group CKD - treated conservatively; HD B - before hemodialysis; HD A - after hemodialysis; PD - peritoneal dialysis; TE B- before kidney transplantation: TE A - after kidney transplantation

Significant differences were found in XOR activity in platelet-poor plasma and in platelets within the groups studied (Fig. 1A, B): the highest mean activity of oxidoreductase was seen in the control group (also in PPP and in PRP), lower before hemodialysis (HD A), and the lowest after kidney transplantation (TE A) (PPP) and in patients treated conservatively (PRP).

**Fig. 2:**
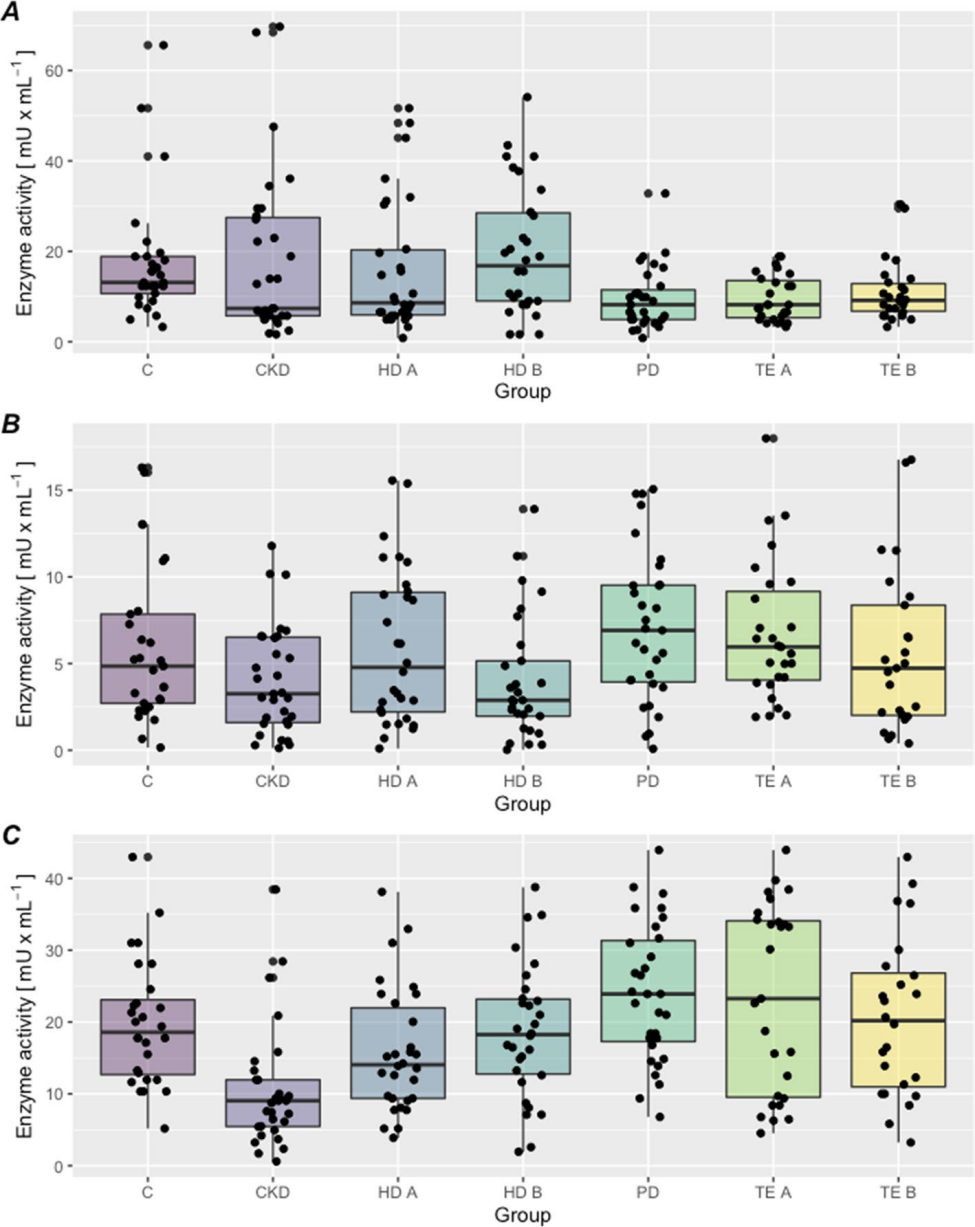
**A, B, C**. The xanthine oxidoreductase isoforms activity in platelet-poor plasma. **A** XD activity, **B** XDO activity, **C** XO activity. C- control group CKD - treated conservatively; HD B - before hemodialysis; HD A - after hemodialysis; PD - peritoneal dialysis; TE B- before kidney transplantation: TE A - after kidney transplantation

Differences in the activity of the XD isoform in the PPP between studied groups were also observed (Table 3). The lowest XD activity was observed in the group of kidney transplant patients, and the highest in the control group (Fig. 2A).

**Table 3:** Activity of XOR isoforms in the platelet-poor plasma, in patients treated conservatively (CKD), hemodialysis patients (before and after - HD B, HD A), peritoneal dialysis (PD), conservative treatment (CKD), before and after kidney transplantation (TE B, TE A) and in the control group (C) (mean ± SD, median – lower and upper quartile)

Activity of XOR isoforms [mU/mL] Groups	XD	XDO	XO
HD B	42,5 ± 14,5 42,7 (10,0; 68,0)	8,7 ± 5,8 8,6 (0,6; 26,2)	67,2 ± 24,5 68,0 (22,6; 122,8)
HD A	45,6 ± 16,8 44,5 (9,6; 83,6)	7,6 ± 4,6 7,8 (0,08; 15,9)	58,5 ± 23,4 57,3 (16,5; 94,6)
PD	34,6 ± 26,1 28,1 (5,2; 116,5)	8,7 ± 6,9 6,8 (0,1; 27,7)	46,9 ± 20,5 43,3 (9,4; 85,0)
CKD	35,8 ± 20,4 37,3 (4,8; 88,5)	19,4 ± 17,5 17,6 (0,9; 75,3)	53,2 ± 72,6 40,4 (1,3; 423,2)
TE B	33,8 ± 16,7 33,3 (7,4; 75,1)	11,3 ± 6,2 10,5 (1,6; 26,0)	40,3 ± 25,2 33,9 (3,9; 124,4)
TE A	22,1 ± 13,8 17,5 (2,2; 53,6)	7,1 ± 3,4 6,7 (1,3; 14,7)	39,1 ± 49,3 28,4 (5,8; 274,0)
C	47,0 ± 15,5 49,2 (16,3; 72,5)	14,8 ± 6,9 14,1 (3,1; 32,0)	58,5 ± 15,4 56,5 (27,1; 86,6)
*P*	<0,001	<0,001	<0,001

There was a statistically significant correlation between the measured XDO activities in the platelet-poor plasma in the groups (Table 3). The lowest activity of the intermediate isoform was demonstrated in patients after renal transplantation (TE A), and the highest in the group of patients treated conservatively (CT) (Fig. 2B).

The relationship between the activity of the XO oxidase isoform oxidase (XO) between the groups in platelet-poor plasma (PPP) (Table 3) approached significance: the lowest XO activity was observed in the group of patients after kidney transplantation and the highest before hemodialysis (Fig. 2C). Other relationships between particular groups are shown in Table 4.

**Table 4:** Statistical differences in the activity of XOR isoforms in PPPs between the studied groups (*p*-value)

**XD in PPP**	
Gropus	***P*** **value**
HD B vs TE B	0,04
HD B vs TE A	<0,001
HD A vs CT	0,044
HD A vs TE B	0,01
HD A vs TE B	<0,001
PD vs TE B	0,031
PD vs C	0,031
CKD vs TE B	0,005
TE A vs TE B	0,004
TE B vs C	0,005
TE A vs C	<0,001
**XDO in PPP**	
Gropus	***P*** **value**
HD B vs CKD	0,002
HD B vs C	<0,001
HD A vs CKD	<0,001
HD A vs TE B	0,014
HD A vs C	<0,001
PD vs CKD	0,02
PD vs C	0,001
CKD vs TE B	0,025
CKD vs TE A	<0,001
TE B vs TE A	0,011
TE B vs C	0,05
TE B vs C	<0,001
**XO in PPP**	
Gropus	***P*** **value**
HD B vs PD	<0,001
HD B vs TE B	<0,001
HD B vs TE A	0,008
HD A vs PD	0,042
HD A vs TE B	0,006
PD vs C	0,016
TE B vs C	0,002
TE A vs C	0,048

**Fig. 3:**
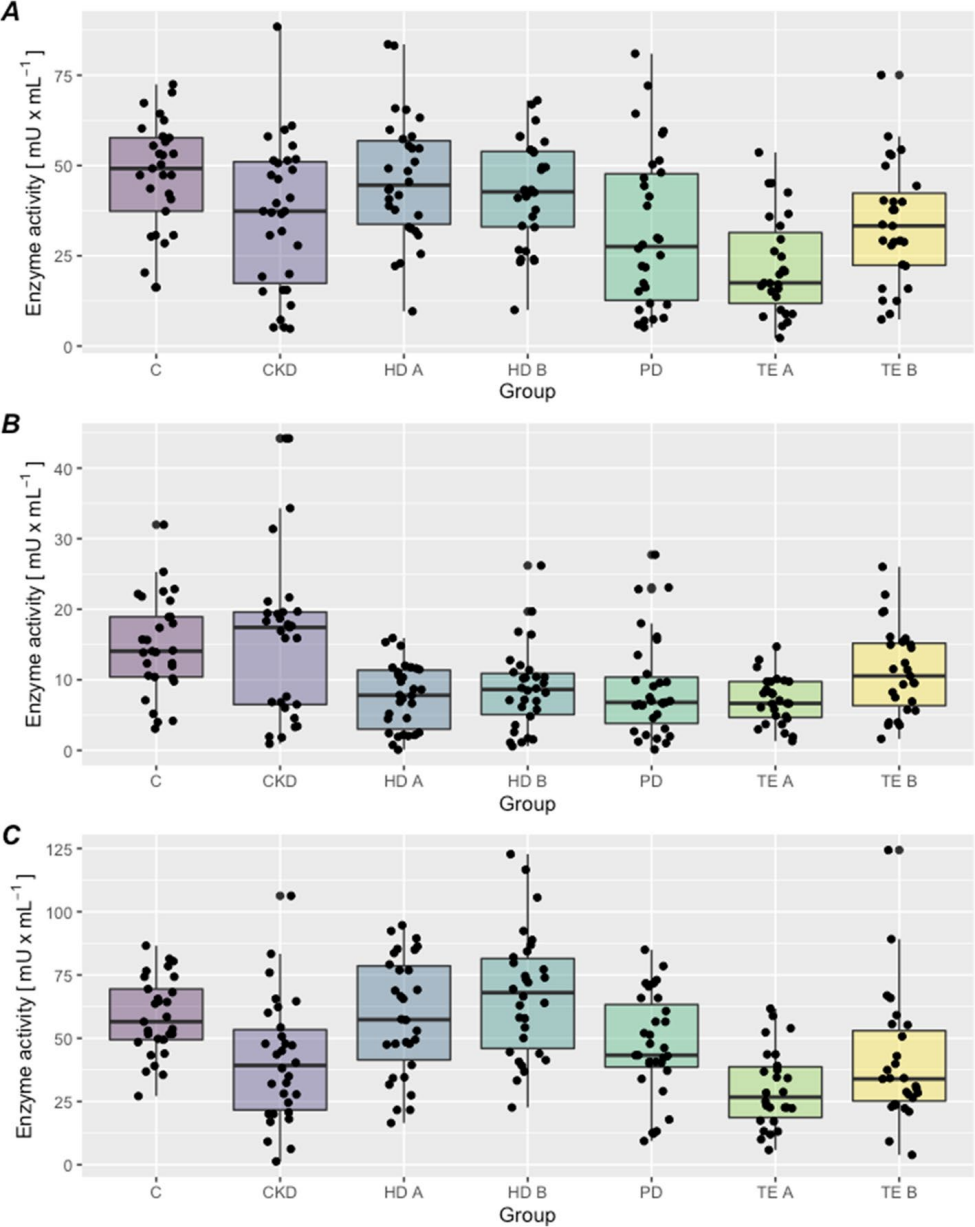
**A,B,C**. The xanthine oxidoreductase isoforms activity in platelet-rich plasma. **A** XD activity, **B** XDO activity, **C** XO activity. C- control group CKD - treated conservatively; HD B - before hemodialysis; HD A - after hemodialysis; PD - peritoneal dialysis; TE B- before kidney transplantation: TE A - after kidney transplantation

A statistically significant difference was found between the activities of XD, XDO, and XO isoforms in the platelet-poor plasma between the examined groups (Fig. 2A, B, C). There was a statistically significant difference in the activity of the XD dehydrogenase isoform in the platelet-rich plasma between groups (Table 5): the lowest XD activity was found in patients treated conservatively, and the highest in patients prior to hemodialysis (Fig. 3A).

**Table 5:** Activity of XOR isoforms in the platelet-rich plasma, in patients treated conservatively (CKD), hemodialysis patients (before and after HD B, HD A), peritoneal dialysis (PD), conservative treatment (CKD), before and after kidney transplantation (TE B, TE A) and in the control group (C) (mean ± OS, median – lower and upper quartile)

Activity of XOR isoforms [mU/mL] Groups	XD	XDO	XO
HD B	19,7 ± 14,3 16,8 (1,6; 54,1)	4,8 ± 5,6 2,9 (0,3; 28,4)	18,6 ± 9,3 18,3 (1,9; 38,8)
HD A	15,9 ± 14,4 8,6 (0,8; 51,7)	6,0 ± 4,5 4,8 (0,1; 15,6)	15,9 ± 8,7 14,1 (3,9; 38,1)
PD	9,6 ± 6,7 8,2 (0,8; 32,8)	7,1 ± 4,3 6,9 (0,08; 15,1)	24,0 ± 9,5 23,9 (6,8; 43,9)
CKD	18,0 ± 18,2 7,4 (1,6; 69,7)	4,0 ± 3,1 3,3 (0,1; 11,8)	10,4 ± 8,3 9,0 (0,6; 38,4)
TE B	13,6 ± 14,8 9,3 (3,3; 79,5)	6,4 ± 6,1 4,9 (0,4; 26,0)	24,7 ± 18,2 21,8 (3,2; 78,2)
TE A	9,6 ± 5,0 8,2 (3,3; 18,6)	6,7 ± 4,0 6,0 (1,9; 18,0)	23,4 ± 13,1 23,3 (4,5; 43,9)
C	17,5 ± 13,6 13,1 (3,3; 65,6)	5,9 ± 4,5 4,9 (0,2; 16,3)	19,4 ± 12,3 19,4 (5,2; 66,5)
*P*	0,008	NS	<0,001

*NS* No statistical significance

There was a statistically significant difference in the activity of the oxidase isoform (XO) in platelet-rich plasma between the study groups (Table 5): the lowest XO activity was found in patients treated conservatively, and the highest in patients before renal transplantation (Fig. 3C; Table 5).

Other relationships between the activity of all XOR isoforms and the type of renal replacement therapy are included in Table 6. Additionally, a statistically significant difference was found between the activities of XD, XDO, and XO isoforms in the platelet-rich plasma between the test groups (Table 6).

**Table 6:** Statistical differences in the activity of XOR isoforms in PRPs between the studied groups (*p*-value)

**XD in PRP**	
Gropus	***P*** **value**
HD A vs HD B	0,022
HD B vs PD	<0,001
HD B vs TE B	0,043
HD B vs TE A	<0,001
PD vs C	<0,001
TE B vs C	0,024
TE A vs C	0,004
**XDO in PRP**	
Gropus	***P*** **value**
PD vs CKD	0,002
CKD vs TE A	0,006
**XO in PRP**	
Gropus	***P*** **value**
HD B vs PD	0,03
HD B vs CKD	<0,001
HD A vs CKD	0,001
HD A vs PD	0,014
HD A vs TE B	0,021
HD A vs TE A	0,011
HD A vs C	0,049
PD vs C	<0,001
CKD vs TE B	<0,001
CKD vs TE A	<0,001
CKD vs C	<0,001

There was also a negative correlation between the activity of XD and XO in PPP and the concentration of uric acid. And also a positive correlation between the activity of a) XD in PPP and XO in PPP, b) XO in PPP, and XD in PRP. A negative correlation was demonstrated between XD activity in PPP and XO activity in PRP. These correlations confirm the physiological importance of oxidoreductase (Table 7).

**Table 7:** Spearman’s rank correlations between the activity of individual XOR isoforms and selected parameters (correlation coefficient; *p*-value)

Parameters	XD in PPP	XO in PPP	XD in PRP	XO in PRP
Uric acid	−0,203; *p* = 0,03	0,276; *p* = 0,008	NS	NS
Creatinine	NS	NS	0,248; *p* = 0,041	NS
XD in PPP	–	−0,562; *p* < 0,001	NS	−0,256; *p* = 0,018
XD in PRP	NS	−0,256; *p* = 0,018	–	NS
XO in PPP	−0,562; *p* < 0,001	–	NS	NS
XO in PRP	−0,256; *p* = 0,018	NS	NS	–

Correlation analysis was performed only among patients treated with renal replacement therapy; XO in PPP-oxidase isoform in platelet poor plasma, XD in PRP - dehydrogenase isoform in platelet-rich plasma, XO in PPP- oxidase isoform in platelet poor-plasma, XO in PRP- oxidase isoform in platelet rich-plasma

There was no correlation between diabetes, BMI, and XOR activity. Dyslipidemia statistically significant influence the activity of XD in PPP (*p* = 0,038) and XO in PRP (*p* = 0,001). The activity of these enzymes was higher in people with dyslipidemia. However, this proves the compensation of oxidative stress and, in our opinion, confirms that dyslipidemia did not have a significant effect on XOR activity.

### The activity of xanthine oxidoreductase isoforms according to gender, duration of dialysis, patient age, cause, and severity of CKD

There was no statistically significant difference in XOR isoform activity between women and men except for the XDO isoform in platelets (Table 8): in men, the activity of this isoform was significantly higher than in women.

**Table 8:** The influence of particular parameters on the activity of xanthine oxidoreductase isoforms

Parameters	XD in PPP	XDO in PPP	XO in PPP	XD in PRP	XDO in PRP	XO in PRP
Gender	NS	NS	NS	NS	0,03	NS
Renal replacement therapy and duration of dialysis	0,002	0,004	<0,001	<0,001	0,012	<0,001
Renal replacement therapy and age	<0,001	<0,001	<0,001	<0,001	<0,001	<0,001
Stage of CKD	<0,001	0,018	**NS**	**NS**	**NS**	0,03
Causes of CKD	0,021	NS	NS	0,01	NS	NS

The table presents *p* values defining statistical significance. The relationship between gender, duration of dialysis, age, stage of chronic kidney disease and the causes of chronic kidney disease and the activity of XOR isoforms was assessed using one-way ANOVA

HD and duration of dialysis - the relationship between the type of therapy (hemodialysis, peritoneal dialysis, patients before kidney transplantation), duration of dialysis and the activity of XOR isoforms

HD and age - dependence between the studied groups (hemodialysis, peritoneal dialysis, conservative treatment, patients before kidney transplantation and control group), patients’ age and XOR isoform activity

The stage of chronic kidney disease - the relationship between the severity of chronic kidney disease based on eGFR and the activity of XOR isoforms

The causes of chronic kidney disease - the relationship between selected causes of chronic disease and the activity of XOR isoforms

*NS* no statistically significant relationship was found

A relationship was observed between the activity of all oxidoreductase isoforms in PPP and PRP plasma, and the type of renal replacement therapy and the duration of dialysis. There were also statistically significant differences between the activity of all tested parameters, and the type of renal replacement therapy and the age of patients (Table 8). The cause of chronic kidney disease was also reflected differences in XD and XO activity in PPP.

There was also a correlation between the activity of XD and XDO isoforms in PPP and the stage of chronic kidney disease (Table 8): XD activity was lowest in Stage 3 disease, and highest in stage 1 disease. The activity of the XDO isoform varied differently: lowest in Stage 3 and highest in Stage 4.

## Discussion

### General Observations

Oxidoreductases, due to its dual activities, is an intriguing and highly important enzyme that allows us to uncover the mechanisms of oxidative stress in the human body. However, the dual activity of XOR also means the interpretation of its functions is more challenging than for other antioxidant enzymes.

In the present study, the highest XOR activity was observed in the control group in PPP and in PRP (second result), meaning that this group is best at oxidative stress compensation. The highest antioxidant isoform (XD) activity observed in PPP and the high activity of the XO isoform confirmed that. This means that the pro-oxidative and antioxidant balance is preserved. Hemodialysis patients before and shortly after kidney transplantation are highly exposed to oxidative stress as a result of the underlying disease as well as the types of renal replacement therapy. This highlights that peritoneal dialysis causes lower exposure of patients to the harmful effects of reactive oxygen species than other renal replacement therapies.

*Dołęgowska et al.* (2010) investigated XOR isoform activity in plasma in individuals after kidney transplantation, divided into three groups: early, slow, and delayed function of the transplanted organ. They showed an increase in XO and XOR activity in all groups after 1 and 5 min after transplantation. XD activity increased in the slow and delayed function groups also after 1 and 5 min. The highest activity was found in the case of the XD isoform (antioxidant) and the lowest in case of the XO isoform (prooxidative). This was probably due to increased oxidative stress as a result of organ transplantation, which the body tried to compensate for by the increased activity of the XD isoform [3, 23].

Increased conversion of XD to XO after kidney transplantation was presented by *Kwiatkowska et al* (2010). During the 6-month observation of patients after transplantation, it was shown that the average level of XOR in serum is constantly increasing from the first day after surgery. This suggests that the XOR level reflects the degree of damages of to the transplanted organ during ischemia/reperfusion (I / R). Further increases in XOR levels can be explained by immunosuppressive therapy, which includes steroids [23–25]. *Herken et al*. (2007) showed that after kidney transplantation, the transformation of the XD isoform into the XO isoform is initiated in the ischemic period and is then continued after reperfusion [25].

In this study, the activity of XOR in PPP after transplantation significantly decreases, similarly with the XD and XDO isoform. The activity of the oxidase isoform also decreases both in the poor and the platelet-rich plasma, but there is not statistically significant relationships. This indicates lower oxidative stress after kidney transplantation because the conversion of the XD isoform to XO is not increased (lower XDO activity after kidney transplantation). These results may indicate small damage to the transplanted organ during the I/R period. Attention should also be paid to the significantly lower activity of XOR and its isoform in patients before and after kidney transplantation in comparison to other forms of renal replacement therapy as well as in comparison to the control group. It suggests a lower efficiency of the antioxidant system in this group of patients.

The optimal type of renal replacement therapy must be selected for each patient to achieve the best treatment outcomes. Many different factors impact on the success of therapy used, one of which is oxidative stress. Therefore, it is crucial to study the effect of dialysis on XOR activity.

In our study, XOR activity decreased after hemodialysis in PPP and PRP. However, it is higher in comparison to the other groups studied (except for control groups). This indicates a higher exposure to oxidative stress in this group of patients. The activity of the XD isoform in the platelet-poor plasma increases after hemodialysis, the XDO isoform decreases, similarly to the XO isoforms. However, these were not statistically significant changes. In turn, in platelets, XD activity is significantly lower after hemodialysis, while XO activity is lower in the HD A group, and the activity of the intermediate isoform is higher after hemodialysis (albeit with no statistical significance). This indicates a low conversion of the antioxidant to prooxidative isoform, which may mean less oxidative stress after hemodialysis.

The influence of the hemodialysis on XOR activity and its isoforms in platelet-poor plasma and platelets was studied by *Cecerska-Heryć et al (2017).* They demonstrated an effect of hemodialysis on the activity of XOR and its isoforms in PPP and in platelets. Besides, they showed a decrease in oxidative stress after hemodialysis, as evidenced by a decrease in XO activity and an increase in XD activity in PPP plasma [26]. This study also confirms the results obtained by other scientists [3, 23–25]. Hemodialysis causes intense oxidative stress, hence the increased conversion of the pro-oxidative to antioxidant isoform after hemodialysis.

On the other hand, *Miric et al*. (2013) showed higher XOR activity before hemodialysis compared to control, and increased XOR activity during renal replacement therapy in patients with GNRI (Geriatric Nutritional Risk Index) ≤ 90 (high risk of complications and mortality due to malnutrition), and a reduction in XOR activity in patients with GNRI>90 (low risk of death due to malnutrition) in which hemodialysis is performed. This may mean that oxidoreductase is involved in hemodialysis-induced oxidative damage, which may contribute to accelerated protein destruction in patients with GNRI ≤90 [27].

*Boban et al.* (2014) showed that total XOR activity was higher in the group of patients suffering from essential hypertension, compared to patients on dialysis. The highest activity in this group was also demonstrated by the XD isoform in relation to the control or dialysis patients. On the other hand, the activity of XO, which mainly contributes to the production of ROS, was the highest in dialysis patients [28].

In the case of peritoneal dialysis, the results achieved by us are ambiguous. In platelet-poor plasma, XD activity is significantly lower than in hemodialyzed patients, as is XO activity. This could indicate a much lower exposure to oxidative stress of patients on peritoneal dialysis than those subjected to hemodialysis. However, the pattern is different in platelets. XD activity is significantly lower in patients from the PD group than in hemodialysis, but XO’s acuteness is significantly higher in peritoneal dialysis patients.

XDO intermediate activity was higher in patients undergoing peritoneal dialysis, which may indicate an increased conversion to the antioxidant isoform. However, it should be remembered that under the influence of RFT there is a leakage of oxidoreductase from cells to plasma. Therefore, the activity of XOR isoforms in platelets related to hemodialysis and peritoneal dialysis may disrupt this picture. During hemodialysis, there is increased activation of platelets accompanied by strong oxidative stress, resulting in ROS can cause XOR to be released to the plasma, which may not happen in the case of peritoneal dialysis: hence, much higher XO and XDO activity in patients from the PD group.

Patients treated conservatively are also exposed to oxidative stress caused by the underlying disease, which can be demonstrated by the highest activity of the XDO isoform and the high activity of the XO isoform in PPP.

### Activity of xanthine oxidoreductase isoforms according to gender, duration of dialysis, patient age, cause, and stage of CKD

Our study revealed no relationship between XOR activity and sex, except for a significant association between XDO activity in platelets. In men, XDO activity was significantly lower than in women. This supports the theory that the activity of antioxidant enzymes may be higher in women. However, the primary purpose of measuring XDO activity is to analyze XD to XO transformations. This confirms the results reported in previous studies, where such a relationship was not observed [19, 25]. Previously, only Decker *et al.* (1982) reported higher XOR activity in male rats compared to females [29].

An age effect was observed in the activity of all isoforms of XOR PPP and platelets in all groups. Patients in groups PD and TE were, on average, younger, and XD and XO activity in platelet-poor plasma was lower in these patients than in the other groups. Only XO isoform activity in platelets was higher in these (younger) groups. These results are different from those obtained for other antioxidant enzymes. This might mean that this group of patients has not yet lost XOR activity under the influence of RFT. However, it is more likely that the type of implemented renal replacement therapy affects XOR activity more than age. This seems to be confirmed by the results of our multivariate regression, as well as studies carried out *by Cecerska-Heryć et al.(2017).*

The relationship between the duration and type of dialysis, and XOR activity has not yet been described in the literature, particularly in the PPP and PRP. However, the issue was raised in the case of other antioxidant enzymes. The reduction of antioxidant enzyme activity in hemodialyzed patients might indicate increased oxidative stress, as observed by *Olszewska et al*. (2004) on the group of patients receiving the same type of renal replacement therapy [30]. *Pawlak et al.(ROK)* observed a decrease in SOD activity and a correlation between the duration of dialysis and superoxide dismutase activity [31]. In another study performed in hemodialyzed children and peritoneally dialyzed children, it was confirmed that dialysis performed causes an increase in oxidative stress, enlargement by the next hemodialysis. Therefore, the longer the dialysis treatment took, the higher oxidative stress was observed [32].

In present study, a significant relationship was found between the duration of dialysis and the type of renal replacement therapy used and the activity of all XOR isoforms in both the platelet-poor plasma and platelets. However, in PPP from longer-lasting dialysis, a decrease in the activity of the XD and XO isoforms was observed. Additionally, the activity of XD, XDO, and XO isoforms increases in the platelets. The XOR activity increased in platelets due to the continuous activation as a result of long-term dialysis and progressive renal disease that causes aggravation of oxidative stress, ROS production, which in turn destroys the blood platelets. However, the lowering of XOR isoforms activity in PPP confirm the results obtained by other researchers on the decrease in antioxidant enzymes activities along with long-term dialysis.

Based on the multivariate regression analysis, it was found that parameters such as the type of renal replacement therapy used, the patient’s age, duration of dialysis, and CKD stage affected the activity of XO in PPP, XD in PRP and XO in PRP, respectively, about 33% of XO in platelet-poor plasma, 39% of XD in platelets and 32% of XO in platelets. (Table 9).

**Table 9:** Analysis of the influence of the tested parameters on the XOR isoforms activity - multivariate regression analysis

Dependent variable	Independent varaible	β	*R*^2^	*p*	*P* for model	F
XO in PPP	Type of renal replacement therapy	-0,59	**0,33**	0,8	**0,004**	2,46
	Age	-0,90		0,89		
	Duration of dialysis	−14,20		0,54		
	Stage of CKD	0,028		0,85		
	CKD causes	−4,25		0,74		
XD in PRP	Type of renal replacement therapy	−1,95	**0,39**	0,38	**<0,001**	3,22
	age	−3,83		0,54		
	Duration of dialysis	−25,10		0,25		
	Stage of CKD	−8,35		0,49		
	CKD causes	−0,16		0,28		
XO in PRP	Type of renal replacement therapy	−4,48	**0,32**	0,08	**0,006**	2,37
	age	−13,20		0,07		
	Duration of dialysis	−26,21		0,3		
	Stage of CKD	0,16		0,09		
	CKD causes	−24,12		0,09		

β - standardized coefficient in the regression equation, R2 - coefficient of determination, *p* - value of the significance coefficient; XO in PPP-oxidase isoform in platelet poor plasma, XD in PRP - dehydrogenase isoform in platelet-rich plasma, XO in PRP- oxidase isoform in plasma

Our study demonstrated the effect of chronic kidney disease on the activity of XD and XO in PPP. The highest activity of XOR isoforms occurred in patients with hypertension and diabetic nephropathy, and the lowest in patients with ADPKD (polycystic kidney disease inherited autosomal dominant). Multivariate regression analysis indicate an independent negative correlation between XO activity in PRP and the severity of chronic kidney diseases.

Due to the fact that XOR plays an important role in the production of uric acid the relationship between the XOR activity and hypertension or cardiovascular risk is widely described. Elevated level of uric acid in the serum are associated with oxidative damage in the vessel wall, inflammatory and proliferative changes of the vessels, hypertension, and impaired renal function. Raised uric acid levels might be a factor of the increased risk of the cardiovascular events. Therefore, the attempts are focused to block the XOR activity to reduce the concentration of UA. The positive effect of inhibition of XOR production on the cardiovascular system was documented [33]. Our study showed a negative correlation between XD activity in PPP and uric acid concentration, and a positive correlation between PPP and UA concentration XO activity. These results confirm the physiological importance of XOR also in the protection against cardiovascular diseases.

*Nakatani et al. (2017)* showed a positive correlation between glucose, uric acid concentration, and XOR activity in patients undergoing hemodialysis. The same study, multivariate regression analysis showed positive, independent correlations between glucose concentration, and type 2 diabetes diagnosed with XOR activity. In contrast, the uric acid concentration correlated positively with XOR activity in hemodialyzed patients with undiagnosed type II diabetes. This study indicate that glycemic control might decrease oxidoreductases-mediated ROS production possibility in hemodialysis patients [34].

The high activity of XO and XD in PPP observed in this study in patients with diabetic nephropathy and hypertension confirm an increased activity of XOR in this type of diseases, due to the distorted level of glucose and uric acid, which may exacerbate chronic kidney disease. For hypertensive patients, high XO and XD activity may be an indicator of an increased risk of cardiovascular events. There is no relationship between the activity of XOR and ADPKD in the literature.

## Conclusion

1. The type of renal replacement therapy used in CKD patients, age of patients, duration of dialysis, CKD causes, and stage of progression significantly affect the activity of XOR and its isoforms.

2. Peritoneal dialysis patients are exposed to less oxidative stress than hemodialysis patients

## Limitations

### Medicines used by patients

Patients in the study group (mainly hemodialysis) took furosemide, which may increase the level of uric acid, a strong antioxidant. It is also the last product in the hypoxanthine degradation pathway, catalyzed by XOR. However, our study showed no correlation between uric acid levels and XOR activity, except for the negative correlation of the XD and XO isoforms in PPP. This correlation only confirms the physiological properties of oxidoreductase; the higher uric acid concentration, the lower XO oxidative isoform activity.

### Kidney transplantation

Initially, we planned to collect biological material from patients after kidney transplantation 5–7 days after transplantation, followed by one month, three months, and six months after transplantation. Unfortunately, due to logistical problems, it was not possible to provide good quality material to our unit at all time points. Therefore, we decided to collect material only approximately 7 days after transplantation.

## Data Availability

The datasets generated and/or analysed during the current study are not publicly available due [these are sensitive data owned by the Pomeranian Medical University in Szczecin] but are available from the corresponding author on reasonable request.
